# U.S. national, regional, and state-specific socioeconomic factors correlate with child and adolescent ADHD diagnoses pre-COVID-19 pandemic

**DOI:** 10.1038/s41598-021-01233-2

**Published:** 2021-11-10

**Authors:** Kesten Bozinovic, Flannery McLamb, Katherine O’Connell, Natalie Olander, Zuying Feng, Sora Haagensen, Goran Bozinovic

**Affiliations:** 1 Boz Life Science Research and Teaching Institute, San Diego Science Center, 3030 Bunker Hill Street, Suite 102, San Diego, CA 92109 USA; 2 University of California San Diego, Extended Studies, 9600 N Torrey Pines Rd, La Jolla, CA 92037 USA; 3 Division of Biology, University of California San Diego, 9500 Gillman Dr., La Jolla, CA 92037 USA

**Keywords:** Neurodevelopmental disorders, ADHD

## Abstract

Attention-deficit/hyperactivity disorder (ADHD), the most diagnosed emerging neurodevelopmental disorder in children, is a growing health crisis in the United States. Due to the potential increase in ADHD severity during and post the COVID-19 pandemic, we analyzed recent national and two state-specific ADHD data distribution among U.S. children and adolescents by investigating a broad range of socioeconomic status (SES) factors. Child and adolescent ADHD diagnosis and treatment data were parent-reported via National Survey of Children’s Health (NSCH). The nationwide childhood prevalence of ADHD is 8.7%, and 62.1% of diagnosed children are taking medication. Louisiana (15.7%) has the highest percentage of children diagnosed with ADHD and California (5.6%) has the lowest, followed by Nevada (5.9%). Multiple correspondence analysis (MCA, n = 51,939) examining 30 factors highlights four areas of interest at the national and state level: race/ethnicity, financial status, family structure, and neighborhood characteristics. Positive correlations between ADHD diagnosis and unsafe school, unsafe neighborhood, and economic hardship are evident nationally and statewide, while the association between a lack of ADHD diagnosis and higher urban neighborhood amenities are evident nationally, but not in two opposing outlier states—Louisiana or Nevada. National and state-specific hierarchical analyses demonstrate significant correlations between the various SES factors and ADHD outcomes. Since the national analysis does not account for the demographic heterogeneity within regions or individual states, the U.S. should rely on comprehensive, county-specific, near real-time data reporting to effectively model and mitigate the ADHD epidemic and similar national health crises.

## Introduction

Attention-deficit/hyperactivity disorder (ADHD), the most common childhood neurodevelopmental disorder, is a growing health crisis in the United States. ADHD is characterized by long-lasting negative consequences, including increased risks for substance abuse and addiction, anti-social and criminal behavior, lower academic attainment, and significantly greater personal and overall economic adversity; an overall estimated incremental median cost of $949.24 per year is associated with ADHD cases, with the average direct expenditure increasing in younger age groups up to preschool age^1–6^. Moreover, childhood ADHD prevalence has been increasing since the late 1990s and early 2000s^7–9^, resulting in an estimated 8.7% (5.3 million) of U.S. children aged 3–17 years diagnosed with ADHD in 2018/2019 [National Survey of Children’s Health (NSCH) 2018–19]. Symptoms in diagnosed adolescents and young adults have worsened during the COVID-19 pandemic, but it is not known if this trend will continue post-COVID-19^10–13^. ADHD outcome data should be promptly reported and analyzed to recognize any potential need for population-specific interventions. Notably, ADHD diagnosis is difficult, and the symptoms are nonspecific^14–16^. Therefore, while current trends indicate a growing number of ADHD cases during the pandemic^10–13^, possible false positive ADHD diagnosis due to increased severity of ADHD-associated symptoms stipulates effective diagnosis and treatment of ADHD.

Based on representative samples of U.S. physician office visits, rates of ADHD diagnosis and medication treatment have been significantly increasing^17–21^. The combined effect of several newly approved ADHD drugs, the 5^th^ edition revision of the *Diagnostic and Statistical Manual of Mental Disorders* manual (*DSM-5*), and updated ADHD guidelines for children, teens, and adults may have contributed to the growing number of ADHD cases^22–24^. The *DSM-5* and American Academy of Pediatrics (AAP) guidelines revised the ADHD diagnosis age criteria by raising the age of symptom onset from seven to twelve years old and outlining pediatric guidelines for children aged 4–18 years^22,24^. Furthermore, the updated *DSM-5* guidelines require adolescents to display only five, instead of six, ADHD symptoms^22^.

National 2016 prevalence estimates indicate that 62.0% of children 2–17-year-old diagnosed with ADHD take medication, accounting for 5.1% of all U.S. children within that age range^25^. The AAP recommends preschool-age children (4–5-year-old) receive behavioral therapy administered by a parent or teacher as the first treatment option, with medication prescribed only if behavioral therapy does not reduce impairment, and that elementary-school-age children (6–11-year-old) and adolescents (12–18-year-old) receive a combination of medication and behavioral therapy^24^. Consistent with the AAP guidelines, the Healthy People 2020 developmental objectives include increasing the proportion of ADHD-diagnosed preschool-age children who receive behavioral therapy, and children and adolescents aged 6–17 years who receive medication, behavioral therapy, or a combination of the two^26^. To evaluate the success of AAP treatment guidelines and Healthy People 2020 goals and to inform effective responses, especially during the potential escalation of ADHD severity during the COVID-19 pandemic, trends related to the treatment of ADHD should be monitored^10–13^.

While regional patterns of ADHD diagnosis and medication prescription vary, the U.S. Southern region generally has higher rates of diagnosis and medication, while the Western region has lower rates of both^9,27^. National data could mask within-state variance relevant to interactions of multiple factors, including state demographics, socioeconomic status (SES) factors, and ADHD diagnosis and medication treatment rates. Targeted studies have identified SES patterns related to ADHD outcomes across counties and greater variation of ADHD outcomes have been reported among counties^28–30^. However, the U.S. lacks a comprehensive system to collect and report ADHD data at the county-level, and the existing data is subject to misrepresentation and discrepancies in sampling and data collection methodology^29–31^.

ADHD diagnosis among children has been correlated to family income, gender, race/ethnicity, primary household language, and insurance status^32–36^. Factors associated with a higher likelihood of taking ADHD medication included being non-Hispanic, living in homes with English as the primary language, living in the South, and having a currently co-occurring condition(s)^25^. These associations have not been reexamined collectively since trends were identified in the 2016 NSCH^25^. While previous studies identified variables associated with a higher likelihood of ADHD diagnosis and medication prescription, many important SES indicators from the NSCH were grouped and not individually assessed in relation to each other, potentially confounding critical trends and associations. Thus, when evaluating available data, such variation within broad SES categories should be considered and identified. This approach likely represents a more equitable survey-based epidemiological data analysis, minimizing generalization biases and potential misrepresentation of individuals’ SES designation.

In this study, we quantify more recent associations between ADHD diagnosis and a broad range of SES factors including income, insurance, area of residence, neighborhood characteristics, race/ethnicity, sex, family structure, household language, parent education, parental nativity, and school environment, utilizing data from the NSCH 2018–2019 dataset. The inclusion of available factors associated with mental health disorders, such as family structure, highlights important associations to childhood ADHD diagnosis in the U.S^37–39^. This comprehensive analysis of both national and representative state-level data differentiates national and state-specific associations.

Since the COVID-19 pandemic is projected to significantly exacerbate the effects of ADHD, specifically in children^10,11^, an analysis of the most recent pre-COVID-19 ADHD data and SES factors is necessary as a basis to better understand during and post COVID-19 ADHD trends^10,40^. Our national and state-specific hierarchical analysis, encompassing relevant SES factors, demonstrates the correlations between the various factors and ADHD outcomes. Since national data does not represent the demographic heterogeneity among states and local regions, we compared the data of two outlier states with opposing ADHD rates to national averages to better understand the effect of SES factors on ADHD diagnosis among children and adolescents in the U.S.

## Results

### Geographic distribution of ADHD diagnosis and treatment

**Figure 1:**
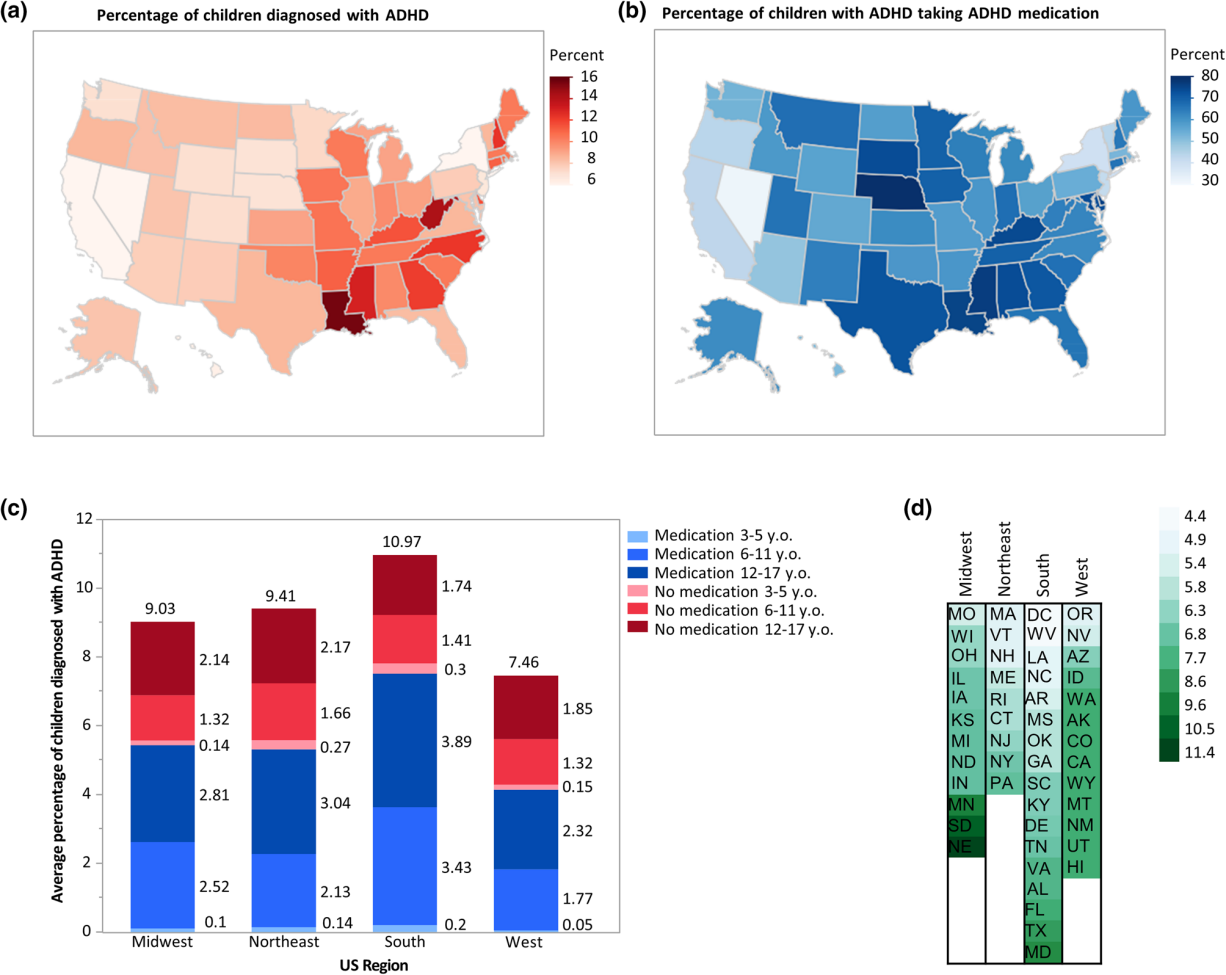
Distribution of parent-reported ADHD prevalence and medication throughout the United States. United States map showing percentages by state of (**a**) children aged 3–17 years currently diagnosed with ADHD and (**b**) children with ADHD currently taking ADHD medication. Darker shades indicate states with relatively higher percentages, and lighter shades indicate states with relatively lower percentages. California (5.6%) has the lowest percentage, Nevada (5.9%) has the second-lowest percentage, and Louisiana (15.7%) has the highest percentage of children who currently have ADHD. Nevada (32.2%) has the lowest percentage, Nebraska (81.76%) has the highest percentage, and Louisiana has the third-highest percentage (76.28%) of children with ADHD currently taking ADHD medication. Percentages of diagnosis and medication were mapped onto the U.S. states using the map function of the graph builder in JMP 14.3.0. (**c**) Compared to the Midwest (n = 11,946) and Northeast (n = 9,102), regional state averages of children diagnosed with ADHD are highest in the South (n = 17,889) and lowest in the West (n = 12,979). Red indicates children diagnosed with ADHD who are not receiving medication. Blue indicates children diagnosed with ADHD who receive medication. Darker shades of both gradients indicate children in older age brackets. (**d**) Cell plot of states ordered lowest (top) to highest (bottom) by region as ratios of the percent of children aged 3–17 years diagnosed with ADHD taking medication to the percent of all children aged 3–17 years diagnosed with ADHD. Darker shades of green indicate higher ratios relative to other states. There are 12 states in the Midwest, 9 states in the Northeast, 17 states in the South, and 13 states in the West. Data were collected from the National Survey of Children’s Health (NSCH) 2018–19 combined dataset.

Prevalence of parent-reported ADHD diagnosis and medication among noninstitutionalized children 3–17-years-old for 2018–2019 are presented by state and region in Fig. 1, with the District of Columbia treated as a state throughout this study. The nationwide prevalence of ADHD is 8.7%, and the percent of children taking medication among those diagnosed is 62.1%. States in the Southern region have the highest statewide average rates of diagnosis (10.97%) and medication (7.52%), while those in the Western region have the lowest statewide average rates of diagnosis (7.46%) and medication (4.14%) (Fig. 1a–c).

Within each region, the oldest age group (12–17-year-old) are the largest group of children diagnosed with ADHD and receiving medication (Midwest 2.81%, Northeast 3.04%, South 3.89%, West 2.32%) and the youngest age group (3–5-year-old) is the smallest in each region (Midwest 0.10%, Northeast 0.14%, South 0.20%, West 0.05%). This pattern is consistent among children diagnosed but not receiving medication, with 12–17-year-old being the largest group (Midwest 2.14%, Northeast 2.17%, South 1.74%, West 1.85%) and 3–5-year-old being the smallest group (Midwest 0.14%, Northeast 0.27%, South 0.30%, West 0.15%). In all regions, more children diagnosed with ADHD are receiving medication than not within the age groups of 6–11 and 12–17. The opposite trend is evident within the youngest age group of 3–5-year-old (Fig. 1c).

When statewide ratios of medication prevalence among children diagnosed with ADHD (Fig. 1b) to overall child ADHD diagnosis prevalence (Fig. 1a) is organized by region; the Midwest ranges from 5.5 in Missouri (MO) to 11.4 in Nebraska (NE), and the Northeast ranges from 5.0 in Massachusetts (MA) to 7.0 in Pennsylvania (PA). In the region with the highest average prevalence of ADHD, the South, the ratios range from 4.4 in the District of Columbia (DC) to 9.1 in Maryland (MD). In the region with the lowest average prevalence, the West, the ratios range from 5.1 in Oregon (OR) to 8.3 in Hawaii (HI; Fig. 1d).

### ADHD diagnosis and treatment variation among representative states

**Figure 2:**
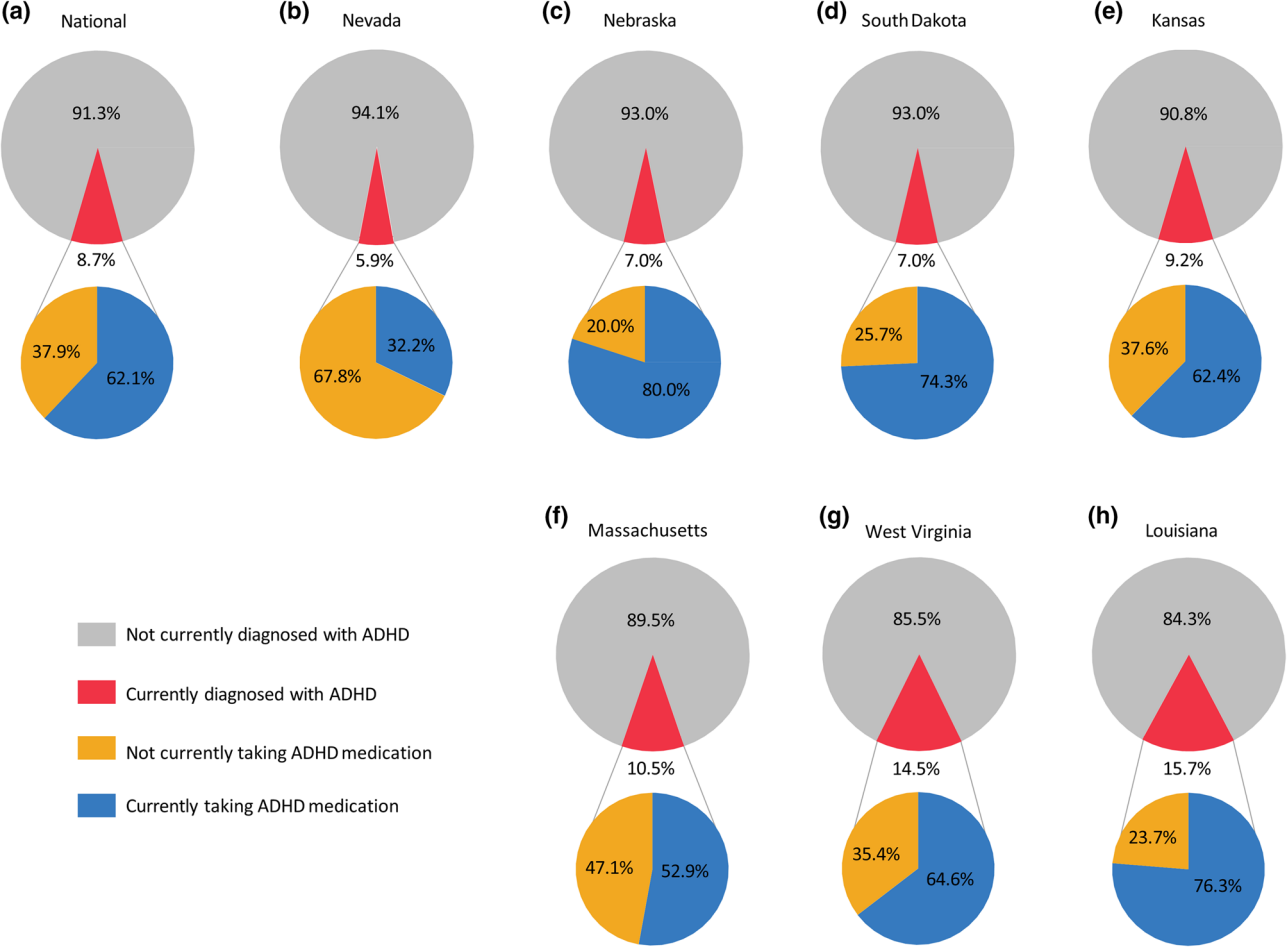
Compared to estimates of (**a**) the national population (n = 51,916), (**b**–**h**) the represented states vary from 5.9 to 15.7% of children age 3–17 years diagnosed with ADHD and 32.2% to 76.3% of children taking ADHD medication among those diagnosed. From lowest to highest rate of diagnosis, the states represented are Nevada (n = 994), Nebraska (n = 953), South Dakota (n = 960), Kansas (n = 1,045), Massachusetts (n = 1,029), West Virginia (n = 1,082), and Louisiana (n = 1,080). Gray indicates children that are not currently diagnosed with ADHD and red indicates children that are currently diagnosed with ADHD. Blue indicates children that are currently diagnosed with ADHD and are taking ADHD medication, and orange indicates children that are currently diagnosed with ADHD but are not taking ADHD medication. (**b**) Nevada represents particularly low rates of diagnosis and medication, while (**h**) Louisiana represents particularly high rates of diagnosis and medication. Data were collected from the NSCH 2018–2019 combined dataset.

Of all states, Louisiana (LA; 15.7%) has the highest percentage of children diagnosed with ADHD and California (CA; 5.6%) has the lowest, followed by Nevada (NV; 5.9%; Figs. 1a, 2). The percentage of children taking ADHD medication among those diagnosed (Fig. 1b) is highest in NE (80.0%), third highest in LA (76.3%), and lowest in NV (32.2%). Kansas (KS) diagnosis (9.2%) and medication (62.4%) rates are closest to the median rates of all states. NE and South Dakota (SD) have low diagnosis rates (7.0% for both), but high medication rates (80.0% and 74.3%, respectively) relative to the median rates. Massachusetts (MA) and West Virginia (WV) have high diagnosis rates (10.5% and 14.5%, respectively), but MA has a low medication rate (52.9%), while WV has a near-median medication rate (64.6%; Fig. 2).

### Multiple correspondence analysis of ADHD diagnosis and SES factors

**Figure 3:**
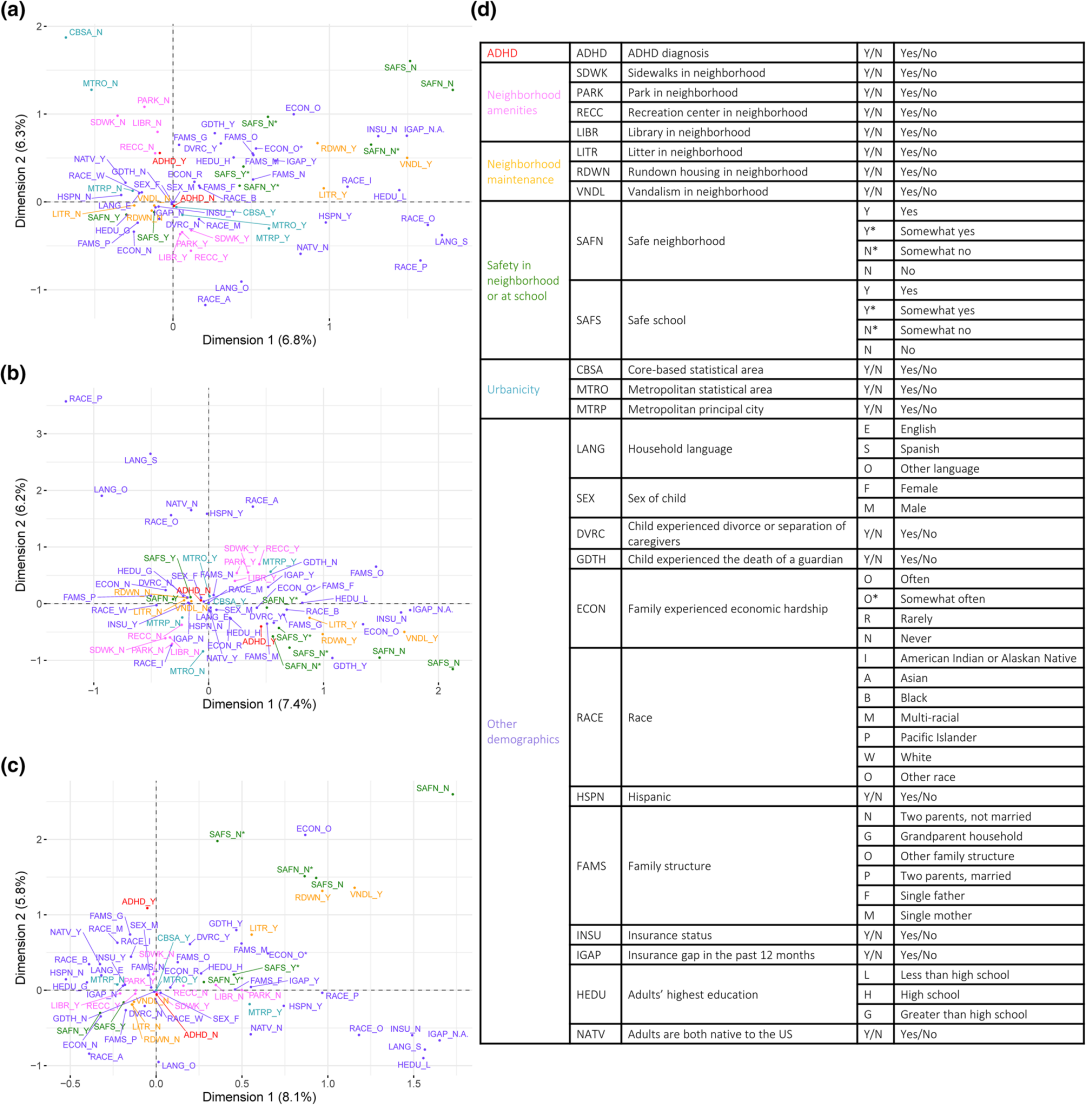
MCAs of ADHD diagnosis, SES factors, and demographics for samples (**a**) nationwide (n = 59,445), (**b**) from Louisiana (n = 1,255), and (**c**) from Nevada (n = 1,154). Data were collected from the NSCH 2018 and 2019 individual responses, and missing responses were imputed using the regularized iterative MCA algorithm of the missMDA R package. (**a**–**c**) MCA plots display associations between variables, with more closely associated variables displayed closer together. For instance, in panel A, ADHD_N and CBSA_Y are closely associated, but CBSA_N and Race_P are not. Positive diagnosis (ADHD_Y) and lack of diagnosis (ADHD_N) are shown in red. The percent variance retained is indicated for each dimension. (**d**) Table of all variables analyzed, with their possible responses and abbreviations.

Individual NSCH responses were analyzed nationally (n = 59,445), within LA (n = 1255), and within NV (n = 1154). Of all analyzed individual response data, 6.7% were missing and imputed for Multiple Correspondence Analysis (MCA; Fig. 3a–c). In MCA, inter-variable proximity indicates the strength of possible associations. The national dataset analysis resulted in 43 dimensions, with inertias ranging from 0.2% to 6.8% and a total inertia of 13.2% for the first two dimensions. LA analysis resulted in 42 dimensions, with inertias ranging from 0.2% to 7.4% and a total inertia of 13.6% for the first two dimensions. NV analysis resulted in 41 dimensions, with inertias ranging from 0.2% to 8.1%, and a total inertia of 13.9% for the first two dimensions.

**Figure 4:**
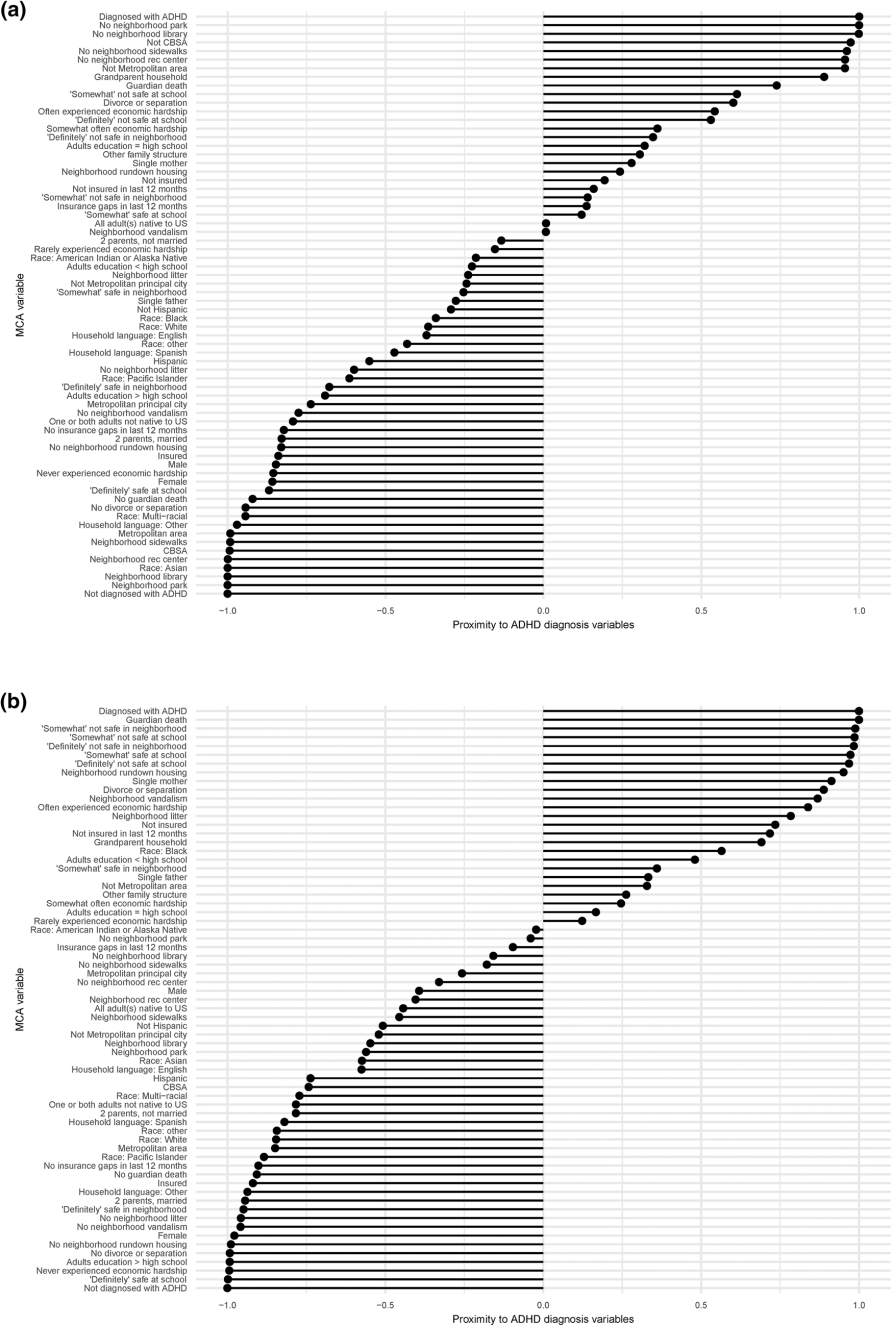

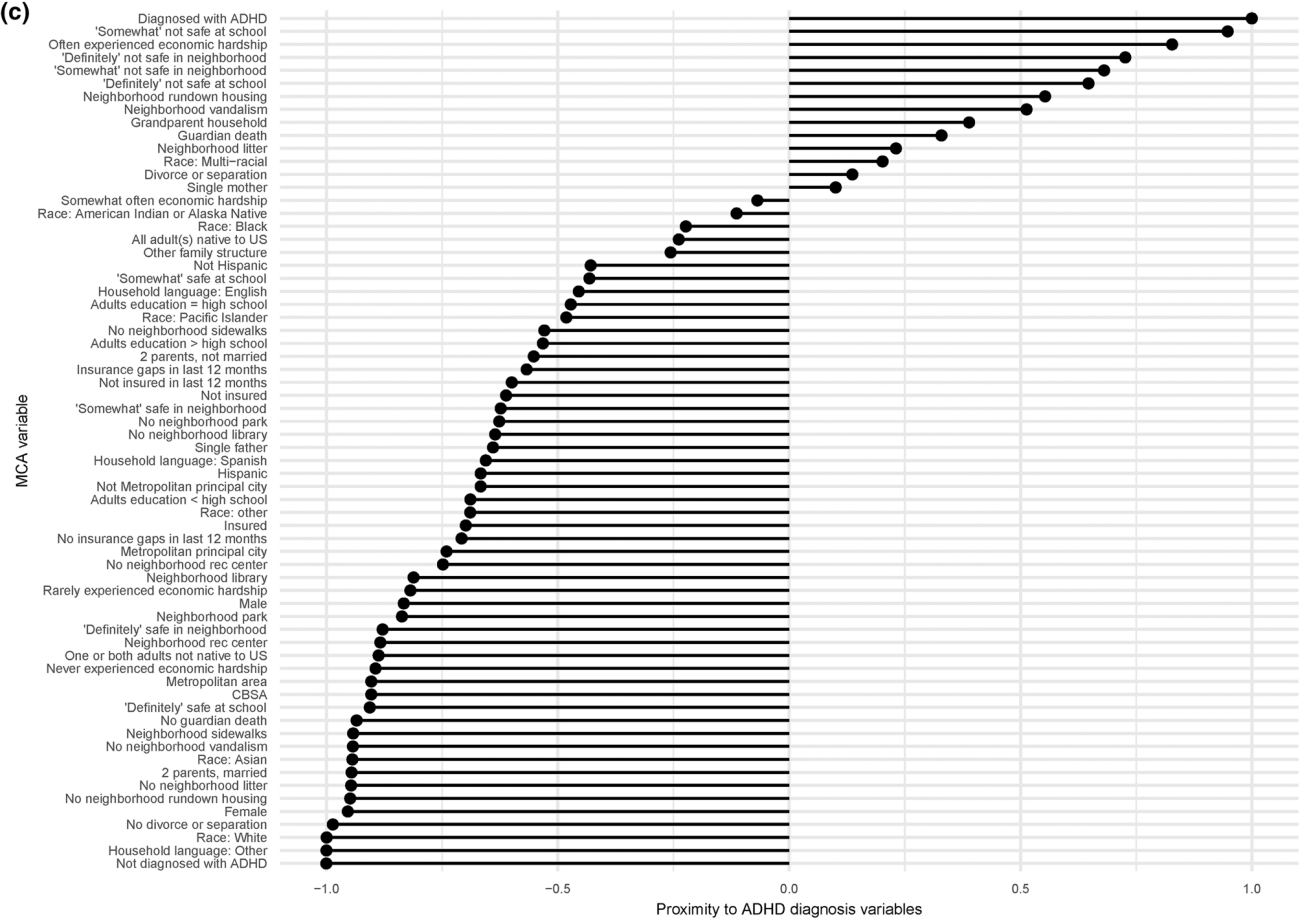
Bar plots representing MCAs of ADHD diagnosis, SES factors, and demographics for samples (**a**) nationwide (n = 59,445), (**b**) from Louisiana (n = 1,255), and (**c**) from Nevada (n = 1,154). Data were collected from the NSCH 2018 and 2019 individual responses, and missing responses were imputed using the regularized iterative MCA algorithm of the missMDA R package. Bars show each variable’s proximity to either diagnosis variable, calculated as (distance to ADHD_N−distance to ADHD_Y)/(distance between ADHD_N and ADHD_Y). A value closer to 1.0 indicates a variable closer to the positive diagnosis and further from the lack of diagnosis, while a value closer to − 1.0 indicates a variable closer to the lack of diagnosis and further from the positive diagnosis.

None of the national, LA, or NV MCAs indicate a relationship with ADHD diagnosis for parental nativity or Hispanic ethnicity (Fig. 4a–c). In all three analyses, unsafe school, unsafe neighborhood, and economic hardship are associated with ADHD diagnosis, while safe school and neighborhood, no economic hardship, two married parents, a household language besides English, no guardian’s death, and no parental divorce or separation are associated with a lack of ADHD diagnosis. All three MCAs display no guardian’s death and no parental divorce or separation in close proximity to a lack of ADHD diagnosis; NV data distribution does not display these adverse experiences near to the positive ADHD diagnosis, although both national and LA MCAs do.

MCA reveals the lack of ADHD diagnosis associated with Asian and multi-racial children in the national dataset, Pacific Islander, White, multi-racial, and other children in LA, and White and Asian children in NV. In LA, a positive ADHD diagnosis is associated with Black children. The national MCA reveals associations between lack of ADHD diagnosis and higher urbanicity [living in a core-based statistical area (CBSA), metropolitan statistical area, or metropolitan principal city], which are not evident for LA or NV (Fig. 3a,d). Neighborhood amenities, namely parks, libraries, recreation centers, and sidewalks, are associated with a lack of ADHD diagnosis nationally, but not in LA or NV. Neighborhood maintenance, namely a lack of vandalism, rundown housing, and litter, show a clear relationship with ADHD diagnosis for LA, but a weaker relationship for NV and almost no relationship nationally. Neither the national nor NV MCAs display proximity between the household adults’ highest education and ADHD diagnosis, but the LA MCA suggests an association between a greater than high school educational attainment and a lack of ADHD diagnosis (Fig. 3a,c). National and LA MCAs display insurance coverage associated with a lack of ADHD diagnosis, while a lack of or discontinuous insurance coverage is moderately associated with ADHD diagnosis. Proximity to ADHD diagnosis is observed for grandparent households in the national dataset and for single-mother households in LA. Only the LA MCA reveals a relationship between sex and ADHD diagnosis, with a lack of diagnosis nearer to female than a positive ADHD diagnosis.

### Correlation of ADHD diagnosis and SES factors

**Figure 5:**
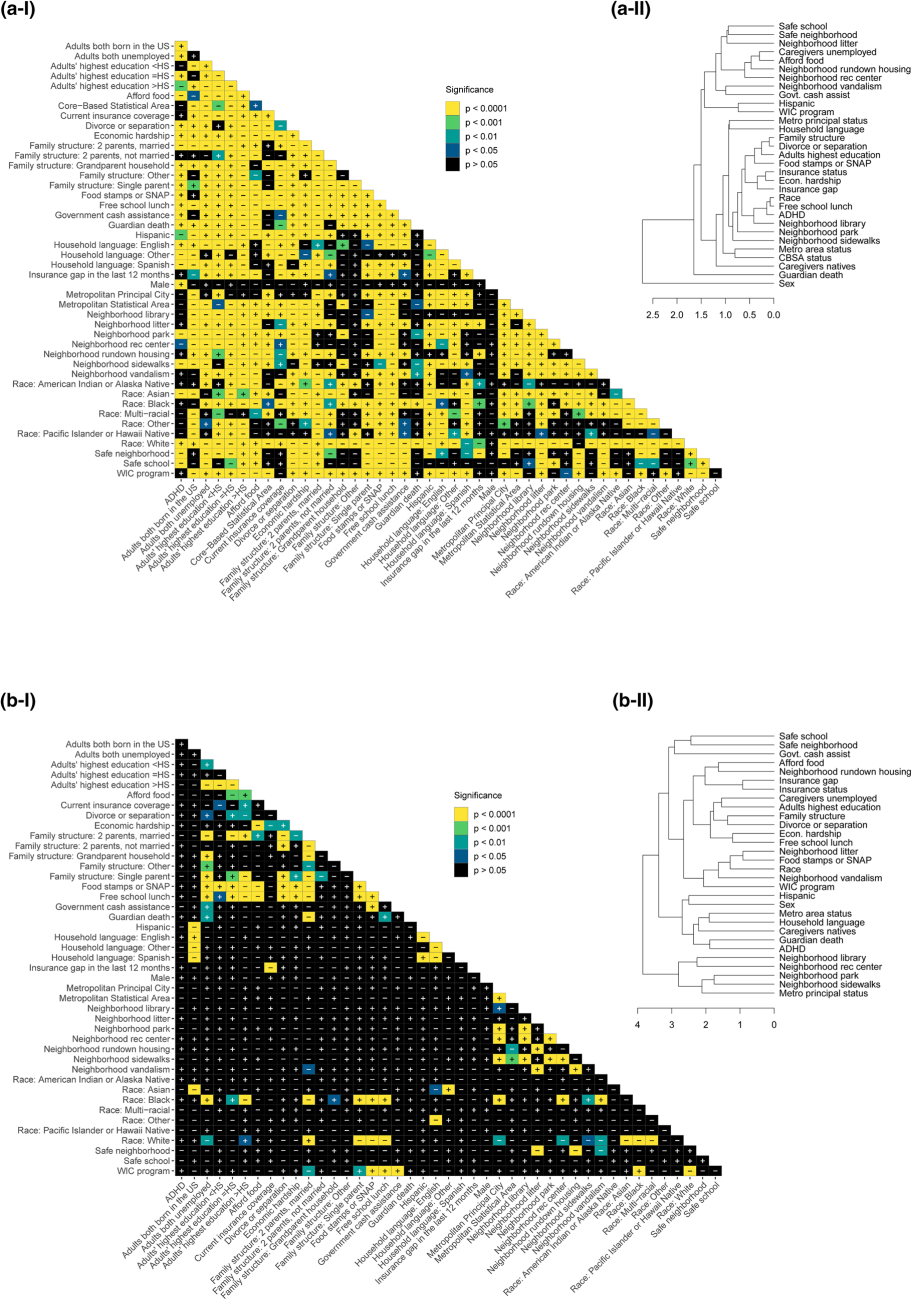

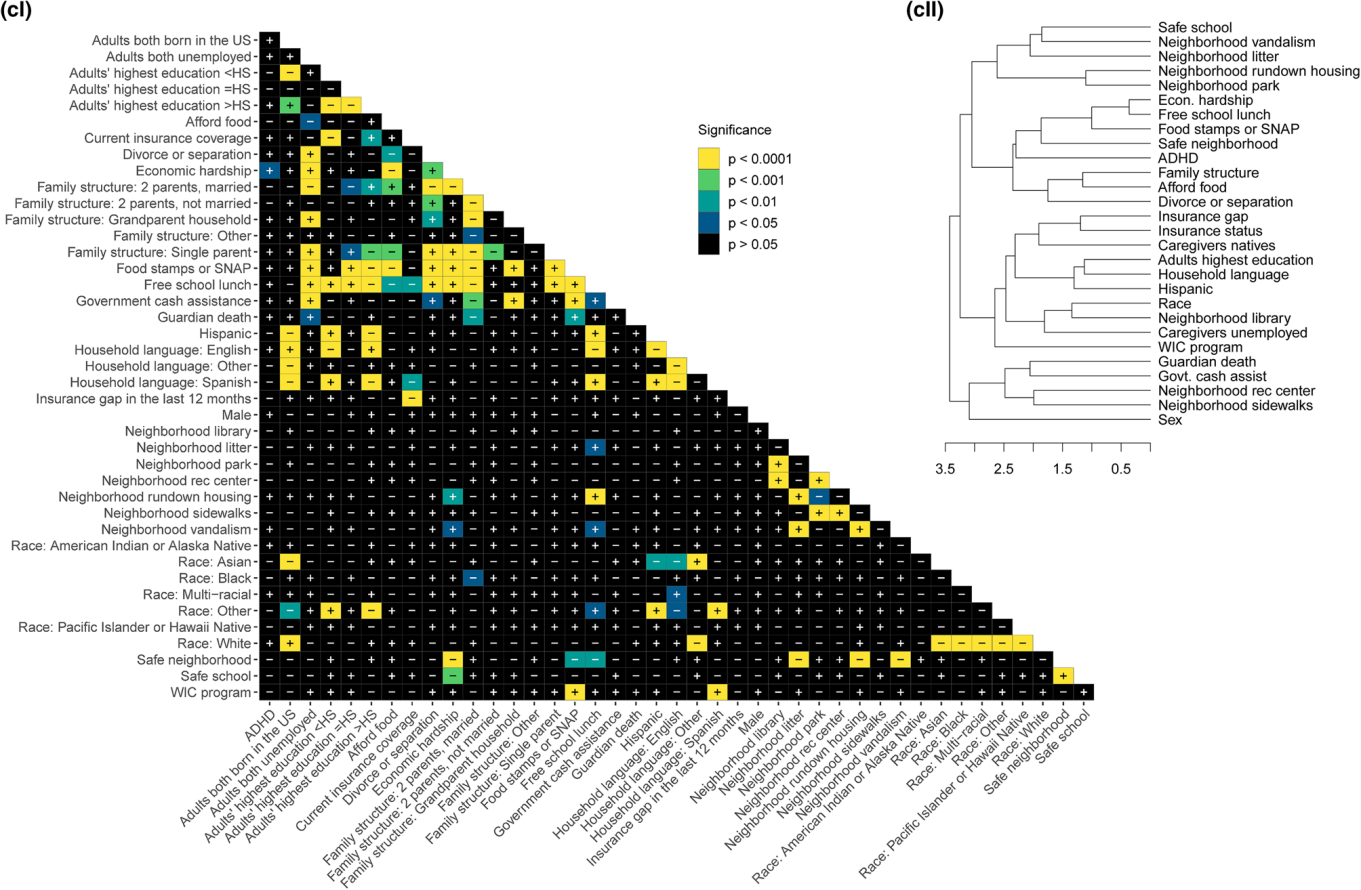
Correlation matrices and hierarchical clustering dendrograms from chi-square test adjusted p-values between ADHD diagnosis, SES factors, and demographics (**a**) nationwide (n = 52,065), (**b**) in Louisiana only (n = 1,084), and (**c**) in Nevada (n = 999) only. In the (I) matrices, black indicates statistical nonsignificance (p > 0.05), darker colors indicate lesser significance, and yellow indicates the greatest statistical significance. “ + ” and “−” indicate positive and negative correlations, respectively. (II) Dendrograms reflect hierarchical clustering of examined variables according to p-values from pairwise inter-variable chi-square analyses. Variables are grouped according to similarities in p-values, and greater branch length along the x-axis indicates greater cluster dissimilarity.

**Figure 6:**
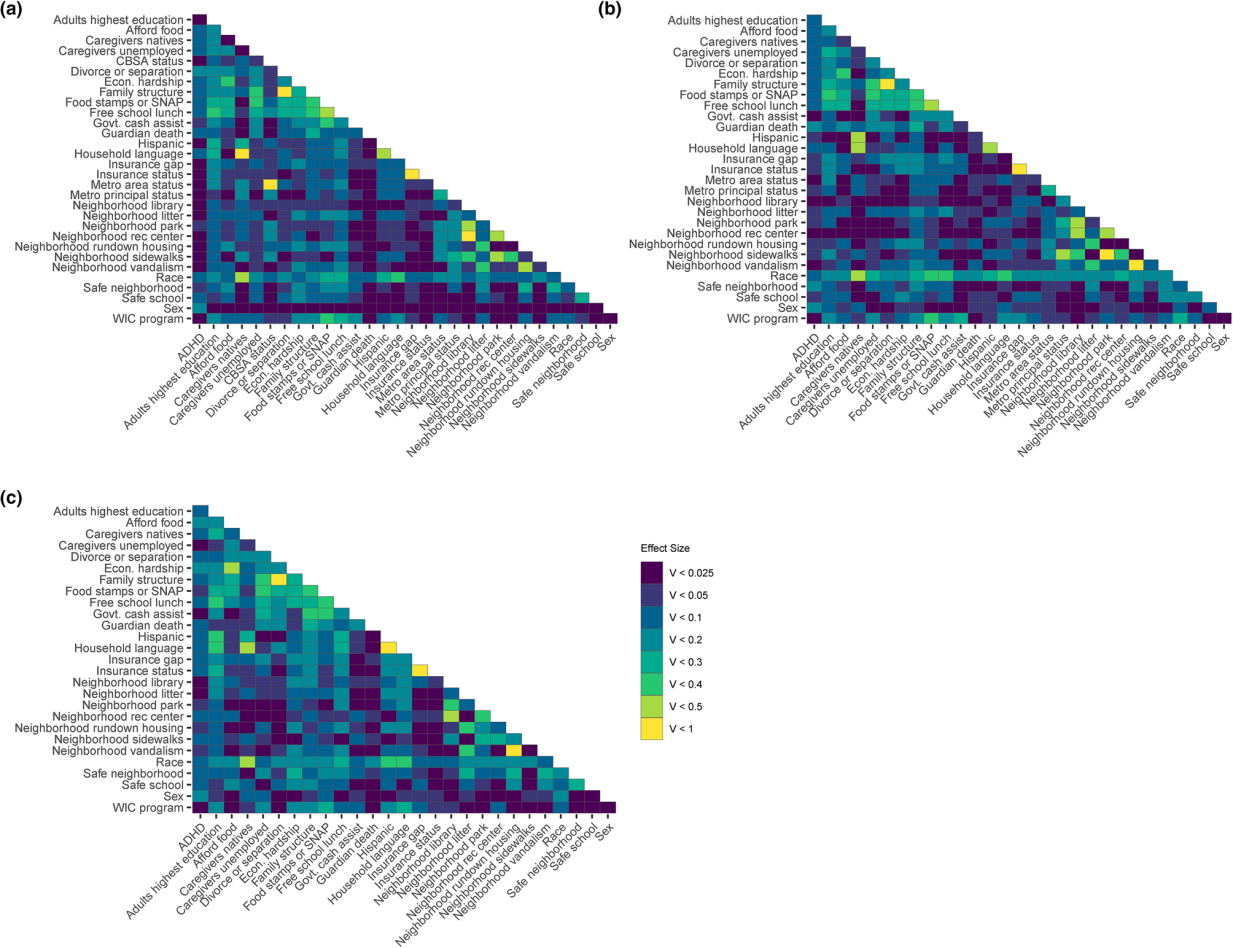
Matrices of Cramér’s V values between ADHD diagnosis, SES factors, and demographics (**a**) nationwide (n = 52,065), (**b**) in Louisiana only (n = 1,084), and (**c**) in Nevada (n = 999) only. Darker colors indicate lower Cramér’s V effect sizes and yellow indicates the highest Cramér’s V effect sizes.

Chi-square and Cramér’s V analyses reveal levels of significance and effect sizes for each response dataset (Figs. 5, 6; Table 1). Nationally, all factors tested are significant against ADHD diagnosis after Bonferroni p-value adjustment except Women, Infants and Children (WIC) program participation, all neighborhood maintenance factors (vandalism, rundown housing, and litter), the presence of a neighborhood library, all urbanicity factors (metropolitan principal city, metropolitan statistical area, and CBSA), insurance status, and insurance gap (Fig. 5a). The largest effect sizes, namely the factors most strongly related to ADHD diagnosis for the national dataset, are sex and having experienced divorce or separation of parents. The smallest effect sizes are the presence of neighborhood sidewalks, the presence of neighborhood recreation centers, Hispanic ethnicity, and the highest educational attainment of household adults (Fig. 6a). Hierarchical clustering of variables based on national dataset p-values (Fig. 5a-II) clusters ADHD diagnosis with free school lunch, and race at the lowest level, which are all then clustered with the presence of a neighborhood library. At the highest level, sex does not cluster with ADHD and the rest of the variables.

**Table 1:** Chi-square and Cramér’s V analyses values of the socioeconomic factors discussed, as compared to ADHD diagnosis.

Region	Factors	*χ*^2^, (df, n)	P-value	Cramér’s V
National	WIC program participation	4.85, (1, 50,783)	> 0.99	0.010
	Vandalism in neighborhood	6.72, (1, 51,011)	> 0.99	0.011
	Rundown housing in neighborhood	15.54, (1, 50,950)	= 0.16	0.017
	Litter in neighborhood	3.18, (1, 50,943)	> 0.99	0.008
	Library in neighborhood	5.13, (1, 50,928)	> 0.99	0.011
	Metropolitan principal city	3.34, (1, 34,850)	> 0.99	0.010
	Metropolitan statistical area	6.97, (1, 36,033)	> 0.99	0.014
	Core-based statistical area	5.53, (1, 23,522)	> 0.99	0.015
	Insurance status	13.09, (1, 51,754)	= 0.60	0.016
	Insurance gap in the past 12 months	6.09e-2, (2, 51,481)	> 0.99	0.012
	Sex of child	675.35, (1, 51,939)	< 0.001	0.114
	Child experienced divorce or separation of caregivers	637.09, (1, 50,511)	< 0.001	0.112
	Sidewalks in neighborhood	31.86, (1, 51,072)	< 0.001	0.025
	Recreation center in neighborhood	18.72, (1, 50,914)	= 0.03	0.019
	Hispanic	28.97, (1, 51,939)	< 0.001	0.024
	Adults’ highest educational	31.94, (2, 51,939)	< 0.001	0.025
Louisiana	Sex of child	16.33, (1, 1081)	= 0.103	0.123
	Safe neighborhood	11.54, (1, 1056)	> 0.99	0.105
	Child experienced the death of a guardian	11.32, (1, 1046)	> 0.99	0.104
	WIC program participation	0.14, (1, 1053)	> 0.99	0.012
	Vandalism in neighborhood	0.018, (1, 1060)	> 0.99	0.004
	Recreation center in neighborhood	0.034, (1, 1057)	> 0.99	0.006
	Library in neighborhood	0.056, (1, 1056)	> 0.99	0.007
	Metropolitan statistical area	0.28, (1, 1081)	> 0.99	0.016
	Government cash assistance	0.034, (1, 1054)	> 0.99	0.006
Nevada	Family experienced economic hardship	17.69, (1, 974)	= 0.046	0.135
	Sex of child	10.33, (1, 994)	> 0.99	0.102
	The ability to afford food	15.91, (1, 974)	= 0.12	0.128
	WIC program participation	2.19e-28, (1, 969)	> 0.99	< 0.001
	Litter in neighborhood	9.99e-4, (1, 975)	> 0.99	0.001
	Library in neighborhood	0.37, (1, 976)	> 0.99	0.020
	Government cash assistance	1.33e-29, (1, 969)	> 0.99	< 0.001
	Caregiver employment status	0.21, (1, 973)	> 0.99	0.015

P-values are adjusted using a Bonferroni correction and considered significant below 0.05.

There are no statistically significant correlations to ADHD diagnosis in the LA dataset (Fig. 5b-I). Besides sex, the largest effect sizes on ADHD diagnosis are neighborhood safety and having experienced the death of a guardian. The smallest effect sizes are WIC program participation, neighborhood vandalism, the presence of neighborhood recreation centers, the presence of neighborhood library, metropolitan statistical area, and family government cash assistance (Fig. 6b). In the LA hierarchical clustering, ADHD is grouped with guardian death at the lowest level, which both share a cluster with U.S. nativity of caregivers, household language, and metro area status.

For the NV dataset, only economic hardship is significant relative to ADHD diagnosis (Fig. 5c-I). Besides economic hardship, the largest effect sizes on ADHD diagnosis are sex and the ability to afford food (Fig. 6c). The smallest effect sizes are WIC program participation, neighborhood litter, the presence of neighborhood library, family government cash assistance, and caregiver employment status. For NV, ADHD is hierarchically clustered with safe neighborhood, food stamps or Supplemental Nutrition Assistance Program (SNAP), free school lunch, and economic hardship.

## Discussion

ADHD is the most diagnosed emerging neurodevelopmental disorder in children and likely an escalating health care crisis during and post-COVID-19 U.S. pandemic. We utilized the most recent publicly available dataset (NSCH 2018–2019) to analyze a broad range of socioeconomic status (SES) factors relative to childhood ADHD and to each other. An analysis of SES factor interactions can influence future data collection strategies and help develop new hypotheses. We selected two opposing outlier states, Louisiana (LA), and Nevada (NV), based on high and low ADHD diagnosis and medication rates, respectively, to compare national to state-specific associations, since the state-level variation of ADHD diagnostic prevalence has been established^27^. While a more comprehensive approach is to analyze the greater variation between and within counties, the data collection systems in the U.S. that report county-specific childhood ADHD diagnosis are limited^29,30^. This analysis is not focused on sex-specific correlations since our chi-square results (Fig. 5a-I) are in agreement with current literature indicating a higher prevalence of ADHD among males^41–44^. While common trends such as higher rates of ADHD diagnosis among adolescents than children are expected, certain weak- to no-associations are surprising: non-significant correlation of recent insurance coverage status (Fig. 5) and limited direct state-specific correlations to ADHD diagnosis^25^.

### Higher ADHD diagnosis and medication treatment rates in the Eastern U.S.

The national prevalence of ADHD in children and adolescents follows previously established regional distribution, with higher diagnosis rates in the Eastern U.S. compared to the West (Fig. 1a)^9^. ADHD medication treatment rates follow a similar pattern like ADHD diagnosis, but are less concentrated regionally, with evident state-specific variations (Fig. 1b): Texas (TX) and Nebraska (NE) have relatively low diagnosis rates, but higher rates of ADHD-diagnosed medicated children, while West Virginia (WV) has a relatively high diagnosis rate but low medication treatment rate. The Western U.S. has the least and Northeastern U.S. has the most variable diagnosis rates (1a); relative to ADHD medication treatment, the Midwestern U.S. is the least variable, while Northeastern U.S. is the most variable (1b). The pattern of ADHD diagnosis by age group is relatively consistent between regions (1c). Considering the ratios of the medication to diagnosis rates, which is arguably the most relevant comparison when assessing the regional distributions, the Midwestern U.S. is the most, while Northeastern U.S. is the least variable (Fig. 1d).

### Regional ADHD medication treatment rates are more variable than diagnosis rates

To better understand ADHD diagnosis associations with SES factors, within-state variation is arguably more informative than either the national or regional distribution, especially since the variation among counties in ADHD outcomes is associated with the socioeconomic composition of the population^27,30^. LA and NV are discussed in detail because each is a national outlier on the opposite ends of the spectrum in their respective regions, marked by the highest and lowest combined average diagnosis and medications rates. Nebraska (NE), South Dakota (SD), Kansas (KS), Massachusetts (MA), and West Virginia (WV) represent in-between state-specific prevalence (Fig. 2). The order of states from NV to LA represents an increasing ADHD diagnosis rate, but the corresponding medication treatment trends do not show a clear pattern related to diagnosis rates. Moreover, while the rates of ADHD diagnosis across states in the Midwestern region (NE, SD, and KS) are consistent (90.8–93.0%), there is considerable variation in medication treatment rates (NE–80%, SD–74.3%, and KS–62.4% respectively; Fig. 2). Notably, although KS is the median state most closely reflecting the national prevalence, the state-specific ADHD data distribution varies and does not necessarily fit national patterns. Within-state variation is consistent with previously identified regional and state pattern discrepancies. Although the state variation determinants are not known, at least two factors, the characteristics of the statewide physician population and laws concerning a schools’ function in ADHD treatment recommendations, are associated with differences in ADHD diagnosis and medication treatment rates^27^. While local physician population and policies regarding schools’ roles in ADHD treatment have potential confounding effects on the variability of ADHD diagnosis between and within states, these data were not included in the NSCH and could not be quantified.

### Older children and adolescents have higher ADHD diagnosis and medication treatment rates

As the cumulative effect of ADHD symptoms results in older children (12–17-year-old) having higher diagnosis rates, regional trends indicate that ADHD-diagnosed adolescents more often take medication than not (Fig. 1c)^25^. Such a tendency is anticipated because medication is more often recommended to patients in this age group as per American Academy of Pediatrics (AAP) guidelines^22,24^. The youngest age group (3–5-year-old) has the lowest diagnosis and medication treatment rates across all regions (Fig. 1c). This is expected, since young children are difficult to diagnose due to a lack of well-defined symptoms in social settings and diagnosis delays, particularly since there is no objective test to diagnose ADHD^27,45–48^. Once diagnosed, the preferred initial treatment for the younger age groups is behavioral therapy^22,24^. Therefore, these results are predictable and indicate a need to investigate SES factors that influence variation in ADHD diagnosis among all age groups.

### Correlation of ADHD diagnosis and race

MCA analysis considers non-linear associations among variables and reveals patterning in complex data sets with categorical dependent variables, while matrices provide statistical correlations between ADHD diagnosis and 30 SES factors nationally and within two outlier states (Figs. 3–6)^49^. The relationships between ADHD and SES factors reveal four overarching categories on the national and state levels: race/ethnicity, financial status, family structure, and neighborhood characteristics. Nationally, ADHD diagnosis is significantly positively correlated with White children and negatively correlated with Asian children (Fig. 5a). Associations of race/ethnicity and SES factors to ADHD diagnosis have generated inconsistent results in the past, which may be due to confounding variables^32,38^. Our analysis supports the association of higher ADHD diagnosis rates among White children and highlights the direct correlation between ADHD and race, accounting for various SES factors^33,34^. It has been documented that race/ethnicity is associated with other SES factors, but the variation of the confounding effects of individual interactions is not as established^50^. The effect size of race to other SES factors indicates the strength of confounding relationships, notably stronger correlations to neighborhood sidewalks, family structure, adults’ highest education, and financial factors including food stamps or SNAP and free school lunch (Fig. 6a).

Race has a higher effect size than other factors nationally and within LA and NV for all variables, except sex and safe schools nationally (Fig. 6). The number of significant correlations and high effect size indicate any relationships between ADHD and race could be due to systemic inequities, especially considering the large racial, ethnic, and cultural diversity which interact with other SES factors^51^. While ADHD diagnosis and race are not significantly correlated within LA and NV (Fig. 4b,c), the implications of race and ethnicity, such as speaking a household language other than English or Spanish, are strongly associated with not being diagnosed with ADHD (Fig. 5a–c); which is expected as English-speaking households have higher rates of ADHD diagnosis^9,34^. Historically, children with special health care needs from non-English primary-language homes lack access to medical homes, a common source of family-oriented primary care^52,53^. Our data shows a significant negative correlation of having current health insurance to a household language other than English or Spanish, which is consistent with previous findings of insurance gaps in non-English primary-language homes^52,54^. National and state-specific overlaps of race and ADHD diagnosis associations highlight the need to better understand both direct and indirect effects of race- and ethnicity-related indicators on ADHD diagnosis and eventual medication trends among children.

### Correlation of ADHD diagnosis and family financial status

We also investigated the relationship between ADHD and indicators of family financial status, including government cash assistance, free school lunches, food stamps/SNAP program, economic hardship, both caregivers being employed, and food affordability. Nationally, all indicators of financial status, except having a gap in health insurance in the last twelve months, are significantly correlated to ADHD diagnosis. This is unexpected because lack of health insurance is an identified factor related to a decreased risk of ADHD diagnosis^34^. Relative to other SES factors, insurance status did not have a direct association with ADHD diagnosis but is significantly correlated to other financial status factors, suggesting an indirect effect. Financial status is significantly correlated to other SES factors associated with ADHD diagnosis. Like race, financial status appears to have a systemic association with ADHD diagnosis, either directly, or indirectly via correlations to parents being divorced or separated, living in a safe neighborhood, and children attending a safe school (Fig. 5a).

Expectedly, financial status indicators are correlated to adults’ highest education and neighborhood amenities, since families with a higher income can afford to live in communities with more resources resulting in better overall health outcomes^55^. Nationally, adults’ highest education level is significantly correlated to every financial status indicator, following the pattern of education higher than high school resulting in better financial outcomes, which is consistent with findings of overall higher education being correlated to lower income inequality^56^. Although not statistically significant for every correlation, that pattern is consistent across LA and NV, as within-state associations relevant to ADHD diagnosis are more variable. Indirect association of ADHD diagnosis could be argued as four indicators of financial hardship (caregivers being unemployed, being on food stamps or SNAP, qualifying for free school lunch, and government cash assistance) are correlated to divorce or separation of parents, which is strongly associated to ADHD diagnosis^39^ (Figs. 4, 5). In NV, ADHD diagnosis is only significantly correlated to economic hardship, although economic hardship correlates to divorce or separation of the parents and family structure, suggesting an indirect correlation^57^. Family structure, specifically divorce/separation and being raised by a single parent, has been reported to increase the likelihood of childhood ADHD diagnosis, reinforcing the existence of correlations between financial status and family structure^38,39^. These findings support the importance of investigating the various interactions between SES factors and ADHD outcomes.

### Correlation of ADHD diagnosis and family structure

Family structure factors, associated with ADHD diagnosis both nationally and within LA and NV, highlight the relationship between the household environment and ADHD diagnosis in children. Family structure, specifically living in a household without two married parents, increases the likelihood of children experiencing economic hardship and being exposed to violence and disruptive households^58^. Adverse childhood experiences (ACEs), including economic adversity and household violence, are reportedly significantly associated with childhood ADHD diagnosis, even when controlling for known parental and child-risk factors of ADHD^59,60^. Hence, family structure is important to investigate in relation to other SES factors and ADHD outcomes. Nationally, grandparent households, the death of a guardian, and divorce or separation are associated with parent-reported ADHD diagnosis (Fig. 4a). Divorce or separation, with the highest effect size (Fig. 6a), is statistically correlated to ADHD and every factor except sex and metro-principal status (Fig. 5a). This reinforces the strong association of living in a household with divorced or separated parents and a higher likelihood of ADHD diagnosis, whether through direct correlation or indirect effects on other SES factors, as well as a lower likelihood of childhood ADHD diagnosis for those living in a two-parent household (Fig. 5a). Therefore, family structure, combined with other relevant SES factors, is a strong marker of ADHD diagnosis nationally and is evident in outlier states: for example, in LA, the death of a guardian is strongly associated with parent-reported ADHD diagnosis (Fig. 4b). Living in a stable home with two married parents results in children with higher standards of living and experiencing fewer stressful events, which is evident in lower rates of ADHD diagnosis among kids^58,61^. The relationship of ACEs and unstable home environments are related to other negative outcomes in young adults, including poor physical and mental health, chronic disease, anxiety, and risk behaviors such as substance abuse which can create a positive feedback loop with symptoms of ADHD^58,62–64^. These associations highlight the importance to better understand the effects of ACEs and family structure on ADHD outcomes.

### Correlation of ADHD diagnosis and neighborhood characteristics

Neighborhood characteristics are strongly associated with ADHD diagnosis in children^65,66^. Nationally, indicators of urbanicity, including living in a core-based statistical area (CBSA), metropolitan statistical area, or metropolitan principal city, are positively correlated with a lack of ADHD diagnosis, which is expected since urban areas have greater access to resources^57^. Neighborhood amenities, including the presence of parks, sidewalks, and recreation centers, promote physical activity which is associated with improved cognition and behavior and could benefit childhood ADHD outcomes^65,67–70^. Lack of neighborhood amenities is strongly associated with ADHD diagnosis (Fig. 4a) and vice versa, while both LA and NV show a distinct pattern of neighborhood maintenance, including rundown housing, litter, and vandalism, more strongly associated with ADHD diagnosis (Fig. 4b,c). These associations are evident in LA, with metropolitan principal city status significantly correlated to neighborhood library, park, recreation centers, and sidewalks access (Fig. 5b). Notably, urbanicity and neighborhood characteristic factors have low effect sizes on ADHD diagnosis highlighting a need for further investigation of their association with ADHD diagnosis (Fig. 6). However, CBSA status in LA and urbanicity factors (CBSA, metropolitan statistical area, and metropolitan principal city status) in NV were suppressed for respondent confidentiality and could not be analyzed.

National and statewide correlations should only be compared with careful consideration of differing sample sizes. Compared to the national analysis, both statewide analyses use a small sample size, resulting in lesser statistical power. Therefore, the overall greater p-values associated with the statewide analyses are expected, given that chi-square tests of smaller sample sizes can yield false negatives, and larger sample sizes can yield false positives. Small sample sizes may also result in less accuracy overall, indicating further needs for proper and comprehensive data collection.

### Conclusions, limitations, and recommendations

Our study highlights important direct and indirect associations between ADHD diagnosis in children and critical SES factors, namely race, financial status, family structure, and neighborhood characteristics. While certain SES factors appear to have strong associations, this analysis is limited by the type of data sourced from the NSCH, representing a cross-section of parent-reported responses^57^. Consequently, we were unable to determine temporal order or causality between SES factors and ADHD diagnosis. While our analysis was limited to ADHD diagnosis and did not account for ADHD treatment differences or diagnosis severity, our focus was on evaluating associations of critical SES factors with ADHD diagnosis to establish a reference for the possible post-COVID-19 increase in ADHD severity among children and adolescents in the U.S.^10,11^. We also limited state-specific analyses to two opposing outlier states and did not account for county-level variation which has been shown to be more variable than among state variation in ADHD outcomes^29,30^. Future studies assessing relationships between SES factors and childhood ADHD diagnosis, severity, and treatment should rely on longitudinal data to establish causality, which is a critical metric for predicting trends and devising effective health care policies.

This report utilizes the most recent publicly accessible NSCH data from a nationally representative sample to highlight associations of 30 SES factors and parent-reported childhood ADHD diagnosis. Although focused on ADHD diagnosis, this report includes important interactions between SES factors, suggesting indirect correlations and confounding factors. This is timely because the COVID-19 pandemic is expected to exacerbate the severity of ADHD outcomes in the U.S.^10,11^. Kazda et al*.* reports pre-pandemic trends in ADHD overdiagnosis in children and adolescents, highlighting the potential for counterproductive, if not harmful, effects on misdiagnosed or mild-symptom-diagnosed youths^16^. Our results may serve as a reference for the post-COVID-19 data, help devise new hypotheses, and highlight gaps within the national data collection system. Data collection and reporting systems for county-level ADHD data are limited in the U.S.^30^ While there are studies addressing the variation between ADHD medication treatment among counties with identified profiles, the medication treatment prevalence data is subject to misrepresentation^29–31^. Moreover, prevalence of ADHD diagnosis is not systematically reported at the county or community level, and studies based on county variation reported discrepancies in sampling and data collection methodology^28,30^. To effectively model and mitigate the ADHD epidemic and similar national health crises, the U.S. should rely on comprehensive, county-specific, near real-time survey and epidemiological data reporting. Such data streams paired with longitudinal data normalized to census numbers are critical components of a primary prevention program development and implementation.

## Methods

All data used represent portions of aggregate parent-reported ADHD diagnosis and treatment data for non-institutionalized children 3–17 years of age, disaggregated into three categorizations: 3–5-year-old, 6–11-year-old, and 12–17-year-old. Data are collected from the annual National Survey of Children’s Health (NSCH) 2018–2019 combined dataset, nationally and by state (https://www.nschdata.org/browse/survey/). NSCH is a United States population-based, cross-sectional survey sponsored by the Health Resources and Services Administration’s Maternal and Child Health Bureau. The NSCH is designed to collect data on health markers of noninstitutionalized children aged 0–17, which has been distributed via an online/mail-based format annually since 2016, previously conducted as a telephone survey every four years. Households that are more likely to contain children are oversampled but are otherwise randomly selected for an invitation household screen. Households with children, as indicated by the screen, receive a questionnaire regarding the health of one randomly selected child. A full explanation of the NSCH methods can be found at https://mchb.hrsa.gov/data/national-surveys/data-user.

Diagnosis and treatment rates were established using either weighted population estimates or survey sample counts, which were both collected directly from the NSCH datasets. The weights for population estimates account for nonresponse, selection probability, household size, household poverty threshold, educational attainment of the respondent, number of children with special healthcare needs in the household, and demographic characteristics of the child. Weighted population estimates and confidence intervals that were not directly provided by the NSCH were calculated from individual response datasets using the provided weights and the xlogit method from the survey package in R 3.6.3^71,72^ following the survey design outlined by the NSCH source and accuracy statement (Table 2).

**Table 2:** Demographics by ADHD status, as counts of the NSCH sample and as estimated percentages of the United States 3–17-year-old population, from 2018–2019.

	Sample count	Ever diagnosed	n = 5886	Currently diagnosed	n = 5416	Sample count	Currently diagnosed and medicated	n = 3476
		%	C.I	%	C.I		%	C.I
All	51,939	9.5	9–10.2	8.7	8.3–9.2	51,916	5.4	5.0–5.7
**Sex**								
Male	27,046	13	12.1–14.1	11.9	11.2–12.7	27,030	7.4	6.8–8.0
Female	24,893	5.8	5.3–6.5	5.3	4.9–5.8	24,886	3.2	2.9–3.6
**Age**								
3–5 years old	9268	2	1.4–2.9	1.9	1.4–2.5	9267	0.8	0.5–1.4
6–11 years old	18,182	9.8	9–10.9	9.3	8.6–10.1	18,171	5.9	5.3–6.5
12–17 years old	24,489	12.8	11.8–14	11.4	10.6–12.2	24,478	7	6.4–7.7
**Race/ethnicity**								
Hispanic	6143	7.3	6–9	6.5	5.5–7.7	6142	3.5	2.8–4.4
White, non-Hispanic	36,010	10.9	10.2–11.5	10.1	9.5–10.6	35,994	6.5	6.1–6.9
Black, non-Hispanic	3373	11.2	9.3–13.5	9.9	8.5–11.5	3368	6.3	5.2–7.5
Asian, non-Hispanic	2522	2.9	1.8–4.5	2.7	1.7–4.0	2521	1.3	0.6–2.5
Other, non-Hispanic	3891	9.6	7.8–11.7	8.8	7.4–10.3	3891	5.1	4.1–6.3
**Family structure**								
Two parents, currently married	35,590	8.1	7.5–8.8	7.4	6.9–8.0	35,580	4.4	4.0–4.8
Two parents, not currently married	3293	10.6	8.2–14	9.2	7.5–11.2	3293	5.7	4.4–7.3
Single parent (mother or father)	9931	11.4	10–12.9	10.5	9.4–11.6	9922	6.6	5.8–7.6
Grandparent household	1679	17.6	13.8–23.6	15.9	13.1–19.2	1677	10.3	8.3–12.8
Other family type	509	20	14.3–28.3	18.6	13.7–24.8	508	14.4	10.1–20.2
Household income (*multiply imputed)								
0–99% FPL	5905	10.9	8.9–12.8	10	8.9–11.3	5896	6.2	5.3–7.3
100–199% FPL	8490	9.8	8.4–11.4	9	7.9–10.1	8484	5.6	4.8–6.6
200–399% FPL	16,197	9.2	8.2–10.5	8.3	7.6–9.2	16,194	4.9	4.4–5.6
≥ 400% FPL	21,347	8.7	8–9.6	8	7.4–8.7	21,342	5	4.6–5.5
**Highest education of adult in household**								
< High school	1324	7.6	5.4–10.9	7	5.2–9.3	1324	4.5	3.2–6.5
High school or GED	6908	11.1	9.5–12.8	10.1	8.9–11.4	6900	6	5.1–7.0
Some college or technical school	12,422	11.3	10.1–12.7	10.4	9.5–11.4	12,415	6.2	5.5–6.9
College degree or higher	31,285	8.5	7.8–9.3	7.7	7.2–8.3	31,267	4.9	4.5–5.3
**Current insurance status**								
Insured	49,354	9.7	9.2–10.4	8.9	8.5–9.4	49,331	5.6	5.2–5.9
Not insured	2400	6.6	4.7–9.1	5.6	4.2–7.3	2400	2.6	1.9–3.6
**Primary household language**								
English	48,387	10.5	9.9–11.2	9.6	9.1–10.1	48,365	6	5.7–6.4
Other than English	3307	3.7	2.4–5.9	3.4	2.3–4.9	3306	1.2	0.6–2.6
**Region**								
Midwest	11,948	10.1	9.2–11.1	9.3	8.4–10.3	11,946	5.9	5.3–6.5
Northeast	9105	8.7	7.7–9.7	7.8	6.9–8.7	9102	3.9	3.3–4.7
South	17,905	11.0	9.8–12.4	10.1	9–11.4	17,889	7.0	6.2–7.9
West	12,981	7.3	6.2–8.5	6.6	5.5–7.8	12,979	3.3	2.4–4.4

GED stands for General Educational Development. *Percent of Federal Poverty Level (FPL) was multiply imputed for analysis.

To determine regional ADHD diagnosis and medication rates by age group, the fifty U.S. states and the District of Columbia were grouped into four regions (Midwest, Northeast, South, and West)^27^. Weighted population estimates of children with ADHD by age group and medication status were calculated into a percentage of the total children aged 3–17 years per state. To examine states in the context of each region, ratios for each state were calculated as the percent of children aged 3–17 years diagnosed with ADHD that are taking medication divided by the percent of children aged 3–17 years that are diagnosed with ADHD. Weighted estimates of ADHD prevalence and medication use among children diagnosed with ADHD nationally and within individual states were used to further examine a variety of states.

Individual household survey responses from the NSCH 2018 and 2019 Topical Data and Input were combined and used to analyze national and statewide associations in ADHD diagnosis and SES factors. Each respondent was weighted to account for the survey year in addition to the same factors used to weight population estimates. The NSCH 2018 Topical SAS Codebook was used to code the questionnaire questions and parent-reported responses (https://www.childhealthdata.org/learn-about-the-nsch/nsch-codebooks). Approximately 71,000 screens were completed for the 2018 NSCH, with 30,530 of the 38,140 qualified households completing the child-specific questionnaire. Approximately 68,500 screens were completed for the 2019 NSCH, with 29,433 of the 36,196 qualified households completing the child-specific questionnaire (https://www.childhealthdata.org/learn-about-the-nsch/methods). Unrelated survey questions were removed before analysis. For multiple correspondence analysis (MCA), missing responses were imputed using the regularized iterative MCA algorithm of the missMDA R package^73^. Missing responses were excluded for chi-square analyses and associated Cramér’s V calculations.

### Data analysis

The data analyzed in this study are from households that completed the child-specific questionnaire and recorded a valid response to the question “has a doctor or other health care provider EVER told you that this child has Attention Deficit Disorder or Attention Deficit/Hyperactivity Disorder, that is, ADD or ADHD?” (n = 9,445). If the parent or guardian responded “yes”, they were directed to answer the following questions:

- Does this child CURRENTLY have the condition?
  - If yes, is it Mild, Moderate, or Severe?
- Is this child CURRENTLY taking medication for ADD or ADHD?
- At any time DURING THE PAST 12 MONTHS, did this child receive behavioral treatment for ADD or ADHD, such as training or an intervention that you or this child received to help with his or her behavior?

ADHD outcomes and SES factors were analyzed at the national, regional, and state levels. Local physician population and policies regarding schools’ roles in ADHD treatment were not included in the NSCH and could not be quantified. Percentages of children with a reported ADHD diagnosis currently taking medication in each state were calculated from weighted population estimates of parent-reported diagnosis and medication use. Percentages of diagnosis and medication were mapped onto the U.S. states using the map function of the graph builder in JMP 14.3.0.

To determine ADHD prevalence within each age group by region, weighted population estimates of each category by the state were used to calculate a percentage out of the total estimated population and then normalized to the total weighted percentage of children with ADHD in that state. Regional percentages are the averages of that category across all states in that region, normalized to the average total weighted percentage of children with ADHD in that region. States selected for further analysis reflect a combination of relatively low or high ADHD diagnosis and medication rates. The two states represent either end of both spectrums, and the states in between represent high diagnosis and low medication rates, low diagnosis and high medication rates, or median medication and diagnosis rates. These data were visualized using the bar graph (Fig. 1c) and cell plot functions (Fig. 1d) of JMP 14.3.0.

To analyze associations among ADHD diagnosis and SES factors, related items from individual household survey responses were analyzed via multiple correspondence analysis (MCA) using the FactoMineR package^74^, then plotted using the Factoextra package in R^75^. Associated bar plots were created using the ggplot2 package, with proximity values calculated as (distance to ADHD_N–distance to ADHD_Y) / (distance between ADHD_N and ADHD_Y). ADHD diagnosis was compared to qualitative survey items related to the family’s SES via Pearson’s chi-square analyses with Bonferroni correction and an adjusted p-value cut-off of p < 0.05 to quantify significance. To quantify the strength of association, Cramér’s V values were calculated for each chi-square value. For items with four possible ordinal responses (e.g. Definitely agree, Somewhat agree, Somewhat disagree, or Definitely disagree), to increase the counts in the contingency tables, the two most positive and two most negative responses were combined to yield two categories of responses (e.g. Definitely/somewhat agree or Definitely/somewhat disagree). To examine similarities in significance between variables, hierarchical clustering based on p-values using the complete linkage agglomerative method was performed in R 3.6.3^72^. Associated matrices were created using the ggplot2 package, software version 3.3.0^76^.

No protocol approval was needed for this study because it uses only publicly available, de-identified data. The author’s institutional review board does not consider the secondary analysis of publicly available data as research on human subjects. All methods were performed in accordance with relevant guidelines and regulations.

## Data Availability

Ethical review was conducted by The U.S. Department of Health and Human Services which administered the National Survey of Children’s Health (NSCH). The NSCH data used in this study are available online on the Centers for Disease Control and Prevention (CDC) website (https://mchb.hrsa.gov/data/national-surveys).
